# Responses of Waveform-Selective Absorbing Metasurfaces to Oblique Waves at the Same Frequency

**DOI:** 10.1038/srep31371

**Published:** 2016-08-12

**Authors:** Hiroki Wakatsuchi, Fei Gao, Satoshi Yagitani, Daniel F. Sievenpiper

**Affiliations:** 1 Center for Innovative Young Researchers, Nagoya Institute of Technology, Gokiso-cho, Showa, Nagoya, Aichi, 466-8555, Japan; 2 Department of Electrical and Mechanical Engineering, Nagoya Institute of Technology, Gokiso-cho, Showa, Nagoya, Aichi, 466-8555, Japan; 3 Wireless Access Research & Innovation, BellLABs, Pudong, Jinqiao, Shanghai, 201206, China; 4 The Graduate School of Natural Science and Technology, Kanazawa University, Kakuma-machi, Kanazawa, 920-1192, Japan; 5 Applied Electromagnetics Group, Electrical and Computer Engineering Department, University of California, San Diego, La Jolla, California 92093, USA

## Abstract

Conventional materials vary their electromagnetic properties in response to the frequency of an incoming wave, but these responses generally remain unchanged at the same frequency unless nonlinearity is involved. Waveform-selective metasurfaces, recently developed by integrating several circuit elements with planar subwavelength periodic structures, allowed us to distinguish different waves even at the same frequency depending on how long the waves continued, namely, on their pulse widths. These materials were thus expected to give us an additional degree of freedom to control electromagnetic waves. However, all the past studies were demonstrated with waves at a normal angle only, although in reality electromagnetic waves scatter from various structures or boundaries and therefore illuminate the metasurfaces at oblique angles. Here we study angular dependences of waveform-selective metasurfaces both numerically and experimentally. We demonstrate that, if designed properly, capacitor-based waveform-selective metasurfaces more effectively absorb short pulses than continuous waves (CWs) for a wide range of the incident angle, while inductor-based metasurfaces absorb CWs more strongly. Our study is expected to be usefully exploited for applying the concept of waveform selectivity to a wide range of existing microwave devices to expand their functionalities or performances in response to pulse width as a new capability.

The advent of metamaterials and metasurfaces, or artificial structures composed of subwavelength periodic units, allowed us to design a wide range of electromagnetic properties including even ones not available from nature^1^,^2^,^3^. These unique properties were applied to significantly extending functionalities of conventional devices and systems (e.g. in antennas^4^,^5^ and absorbers^6^,^7^,^8^,^9^,^10^) or developing new kinds of electromagnetic applications (e.g. cloaking devices^11^,^12^,^13^, superlenses^14^,^15^ and wavefront conversion^16^,^17^,^18^). Additionally, designing these artificial structures with nonlinear media or nonlinear circuits gave us an additional degree of freedom to control electromagnetic properties^19^,^20^,^21^,^22^. Especially, metasurfaces were recently designed with several circuit elements including schottky diodes so that they enabled us to sense difference in the waveforms of incoming waves or pulse widths^23^,^24^,^25^ (Fig. 1). This new capability to distinguish different waves even at the same frequency was expected to give us another degree of freedom to control electromagnetic waves, thereby leading to development of new kinds of microwave devices and applications such as waveform-selective wireless communications^26^. However, all the past studies were evaluated with only surface waves or free-space waves at a normal angle^23^,^24^,^27^, although in reality electromagnetic waves scatter from various structures or boundaries and therefore illuminate such metasurfaces at oblique angles. For this reason we clarify angular dependences of waveform-selective metasurfaces both numerically and experimentally. Especially, this study focuses on two types of waveform-selective metasurfaces, specifically, capacitor-based waveform-selective metasurfaces and inductor-based waveform-selective metasurfaces, each of which more effectively absorbs short pulses and long pulses, respectively, at the same frequency^26^. This performance remains unchanged for a wide range of incident angle but becomes reduced for a large incident angle, which is also discussed to improve in this study. Our results demonstrated here are expected to open up possibilities to apply the waveform selectivity for a wider range of electromagnetic applications even with angled waves.

Although the fundamental absorbing mechanism of waveform-selective metasurfaces is fully described elsewhere^23^,^24^, waveform selectivity is realized in the following manner. The metasurfaces are composed of periodic conducting square patches (copper, 17 × 17 mm^2^ with minor crops) and ground plane (PEC, namely, perfect electric conductor) as well as a dielectric substrate (Rogers3003, 1.5 mm thickness) in between. Additionally, several circuit components are deployed between the conducting patches as illustrated in the left of Fig. 2 (see blue and green). Under these circumstances a set of four schottky diodes (Avago HSMS-2863/2864, blue here) plays the role of a diode bridge. For this reason an incoming wave induces electric charges on each patch, which are fully rectified to an infinite set of frequency components but mostly to zero frequency. This energy flow is controlled by exploiting the time-domain responses of capacitors and inductors (the green in the left of Fig. 2), i.e. capacitors store the energy and gradually build up their electric potentials, while inductors block the incoming energy due to the presence of electromotive force during an initial time period. These two circuit elements are combined with resistors in parallel and series, respectively, so that the energy stored in the capacitors is fully dissipated unless the incoming pulse is too long, which fully charges up the capacitors. In contrast, inductor-based waveform-selective metasurfaces do not absorb short pulses, although a long enough pulse is strongly absorbed as the electromotive force is reduced due to the zero frequency component. We have also demonstrated other types of waveform-selective metasurfaces, for instance, to absorb or transmit pulses of a defined width^26^. The conclusion obtained in the following study, however, is expected to remain the same if they are designed in a proper manner. Additionally, it is very important that our structures do not exploit the difference in frequency spectrum that generates due to the discontinuity of a pulsed sinusoidal signal at both of the beginning and end^28^. Although an extremely short pulse contains not only the oscillating frequency component but also a wide range of other frequencies, we test these structures only with long enough pulses. In other words the bandwidths of the sine wave pulses used in this study are narrow enough compared to those of our structures.

To model the structures, we used a co-simulation method that integrates an electromagnetic simulator (HFSS version 2014) with a circuit simulator (Designer version 2014) as our previous studies^23^,^26^. In this method a periodic unit of a waveform-selective metasurface was modelled with periodic boundaries in the electromagnetic solver so that it effectively represented an infinite array of the periodic structure. Importantly, this electromagnetic model replaced all the circuit elements with lumped ports that are connected to the actual circuits in the circuit simulator where all the results were obtained. Therefore, this method gave us effectively the same results as ones obtained from electromagnetic simulators including all the circuit components but with a significantly reduced simulation time, which contributed to readily sweeping simulation parameters such as frequency, power, circuit values, etc at the expense of visualizing field distributions in the analysis space^29^. All the other information including structure dimensions is given in Fig. 2.

Measurement samples were prepared to have the same structure dimensions and material properties as the ones used for the simulation other than the entire board sizes that were 270 × 270 mm^2^ (corresponding to 15 × 15 units) (see the right of Fig. 2). Compared to our past studies^23^,^26^,^27^, we modified and improved our measurement system for conducting oblique incidence measurements in an efficient manner. For example, incoming signals were generated from a vector network analyzer (VNA) (Keysight Technologies N5249A) that, compared to signal generators (which are used for most conventional pulse measurements), significantly reduced the total measurement time but still allowed us to tune the pulse width. These signals were then amplified by an amplifier (OPHIR 5193RF) so that waveform selectivity was observed with a large enough power even in free space. After the amplifier an isolator (Pasternack PE8311) was used to avoid strong reflection. Two of couplers (ET Industries C-058-30 and Krytar 102008010) were also added to monitor and control the incoming power level. Additionally, a pair of standard horn antennas (Corry Micronics Inc. CMILB-284-10-C-S) was used to radiate out the pulsed signals to a surface under test (SUT) and receive the reflected waves that were eventually sent back to the VNA through the second port. Moreover, some attenuators (Weinschel Associates WA24-10-12, WA27-20-21 and WA27-30-21) were connected to part of our measurement system to protect instruments. Note that our measurements required to properly set the input power level at the location of the SUT unlike most metamaterial absorber measurements that do not involve nonlinearity and thus simply observe a relative power. For this reason the power density at the SUT was first estimated by replacing the SUT with an additional horn antenna that was connected to a set of a power sensor and power meter (Anritsu MA2411B and ML2496A). The power density *W*_*t*_ was calculated from *W*_*t*_ = *P*_*r*_/*A*_*r*_^30^ where *P*_*r*_ is the power received at the second horn antenna. *A*_*r*_ represents the effective area of the antenna, which is obtained from the physical aperture size *S* (0.143 × 0.103 m^2^) multiplied by the total antenna efficiency *e*. Here *e* was simply estimated from VSWR (voltage standing wave ratio) and scattering parameter *S*_11_, namely, 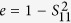 and *S*_11_ = (VSWR-1)/(VSWR + 1). Although more precise measurement can be performed by taking account of the frequency dependence of the gain and *S*_11_ of the antennas used, we simply adopted the values at 4.0 GHz for all the frequencies for the sake of simplicity.

## Results

**Figure 3:**
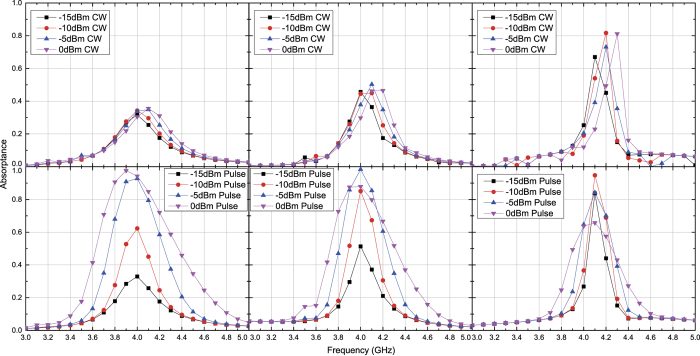
Simulated absorptance of capacitor-based metasurface for TE polarized waves. The incident angle was set to 20 (left), 40 (centre) and 60 degrees (right). The top and bottom panels respectively represent the absorptances for CWs and short pulses (50 ns long). See Supplementary Information for the difference between CW and pulse absorptances.

**Figure 4:**
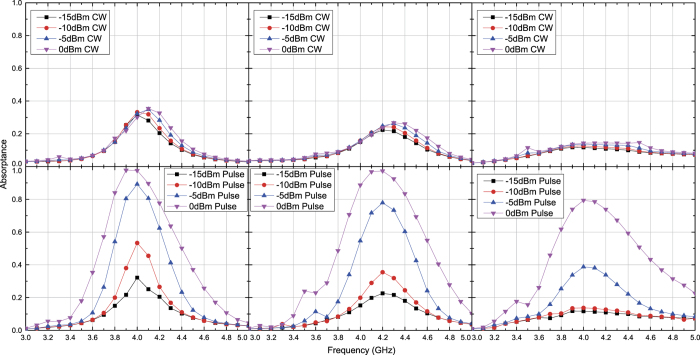
Simulated absorptance of capacitor-based metasurface for TM polarized waves. The incident angle was set to 20 (left), 40 (centre) and 60 degrees (right). The top and bottom panels respectively represent the absorptances for CWs and short pulses (50 ns long). See Supplementary Information for the difference between CW and pulse absorptances.

**Figure 5:**
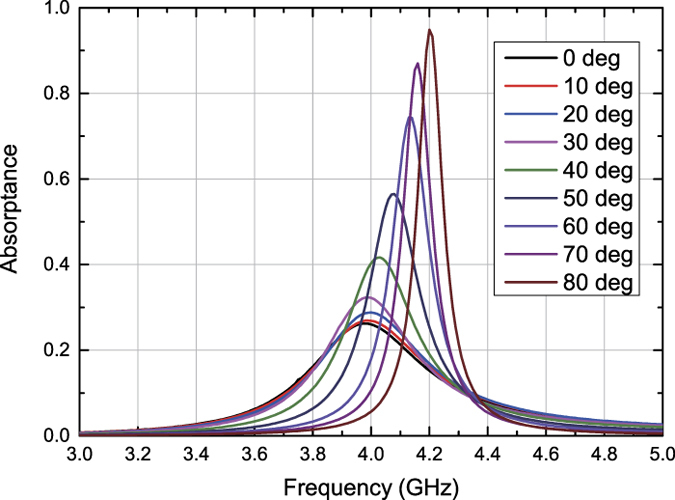
Simulated absorptance of capacitor-based metasurface for low power TE polarized waves. The structure simulated here is the same model as that of Fig. 3.

**Figure 6:**
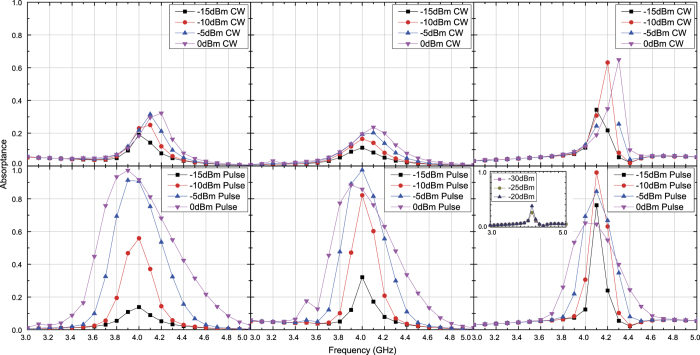
Simulated absorptance of capacitor-based metasurface for TE polarized waves with *R*_*s*_ reduced from 6 to 0 Ω compared to Fig. 3. The incident angle was set to 20 (left), 40 (centre) and 60 degrees (right). The top and bottom panels respectively represent the absorptances for CWs and short pulses (50 ns long). The bottom right panel contains absorptances for lower power levels.

**Figure 7:**
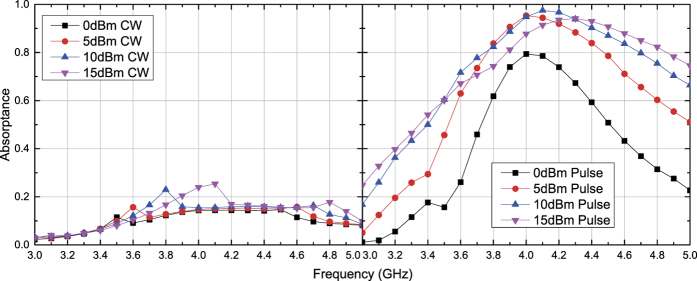
Simulated absorptance of capacitor-based metasurface for TM polarized waves. The incident angle was set to 60 degrees, and the input power was increased to 15 dBm at maximum compared to the right panels of Fig. 4.

Under these circumstances capacitor- and inductor-based waveform-selective metasurfaces were tested numerically and experimentally with transverse electric (TE) waves and transverse magnetic (TM) waves. Figures 3 and 4 plot simulation results of the capacitor-based metasurfaces. It turns out from these figures that as seen in normal incidence^27^, there appears a clear difference between absorptance of 50 ns short pulses and that of CWs, when the input power and incident angle were respectively set to 0 dBm and 20 degrees, which is a relatively small angle. Regarding the TE polarization, increasing the incident angle led to reducing the absorptance of short pulses and enhancing that of CWs, thereby resulting in poor waveform selectivity. In fact, these absorptances approach that of a low power simulation result, which is demonstrated in Fig. 5. Also, note that the larger the incident angle is, the more the wave behaves like a TE surface wave. In this case the wave does not have a strong electric field component due to the presence of the ground plane (PEC), which presumably weakened the rectification process of diodes. As a consequence, the absorption seen in this case results from the loss in the substrate as well as that in diodes (i.e. the intrinsic series resistive component working for low power signals). For example, reducing the series resistance *R*_*s*_ of the diodes from 6.0 Ω to ideally 0.0 Ω improves the waveform-selective response as shown in Fig. 6. Another point noticed here is that the peak absorptance level for the short TM wave pulses tends to be suppressed to a lower value by increasing the incident angle. However, it is important to note that this situation approaches a TM surface wave as well. According to our previous study^26^, a stronger level of input power was needed to achieve a nearly perfect absorption for TM surface waves. Specifically, this study maximized performance of the same capacitor-based metasurface by using a 15 dBm input power level inside a TEM waveguide that was 22 mm tall and 18 mm wide, which means that the power density is approximately 79.9 *μ*W/mm^2^. In our study, however, 0 dBm at 60 degrees is only 1.5 *μ*W/mm^2^. This is why the absorptance can be enhanced by more increasing the input power as demonstrated in Fig. 7, where the same capacitor-based metasurface model was simulated with up to 15 dBm input power (which corresponds to 48.8 *μ*W/mm^2^).

**Figure 8:**
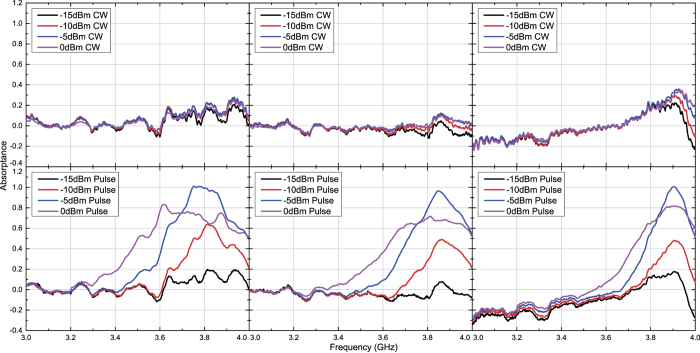
Measured absorptance of capacitor-based metasurface for TE polarized waves. The incident angle was set to 20 (left), 40 (centre) and 60 degrees (right). The top and bottom panels respectively represent the absorptances for CWs and short pulses (50 ns long). See Supplementary Information for the difference between CW and pulse absorptances.

**Figure 9:**
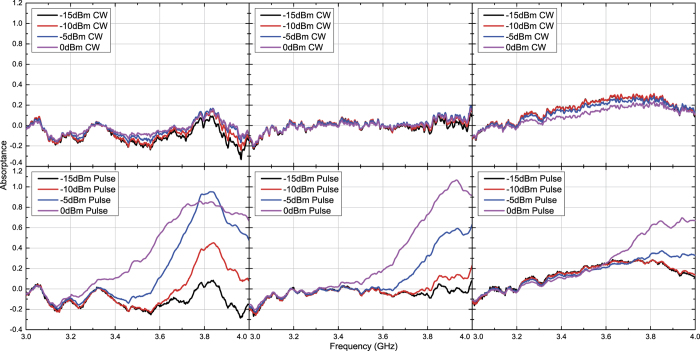
Measured absorptance of capacitor-based metasurface for TM polarized waves. The incident angle was set to 20 (left), 40 (centre) and 60 degrees (right). The top and bottom panels respectively represent the absorptances for CWs and short pulses (50 ns long). See Supplementary Information for the difference between CW and pulse absorptances.

The measurement results seen in Figs 8 and 9 also show similar results with the simulation results except for some minor differences. For instance, the power level to maximize absorptance was reduced from 0 dBm to −5 dBm at 20 degrees for both polarizations. This is due to multiple factors including the simplified total antenna efficiency, which was calculated only from the reflection coefficient and thus led to the underestimate of the measured power. Despite these differences, however, the measurement results still showed similar results with those seen in the simulation and demonstrated that waveform selectivity is realizable even with oblique waves.

**Figure 10:**
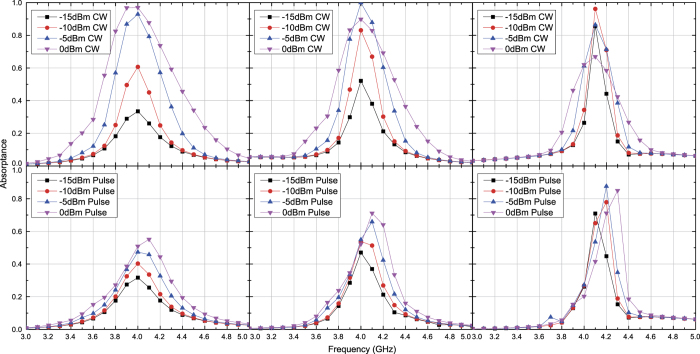
Simulated absorptance of inductor-based metasurface for TE polarized waves. The incident angle was set to 20 (left), 40 (centre) and 60 degrees (right). The top and bottom panels respectively represent the absorptances for CWs and short pulses (50 ns long). See Supplementary Information for the difference between CW and pulse absorptances.

**Figure 11:**
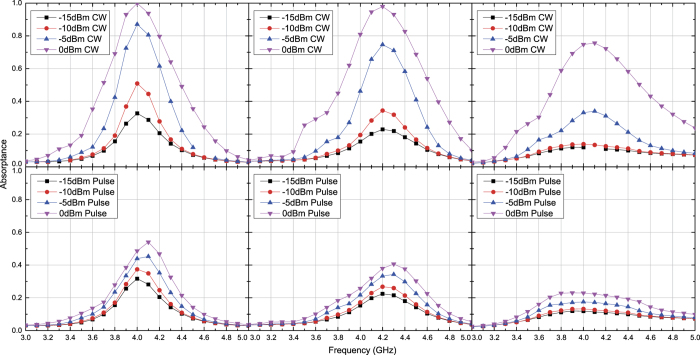
Simulated absorptance of inductor-based metasurface for TM polarized waves. The incident angle was set to 20 (left), 40 (centre) and 60 degrees (right). The top and bottom panels respectively represent the absorptances for CWs and short pulses (50 ns long). See Supplementary Information for the difference between CW and pulse absorptances.

**Figure 12:**
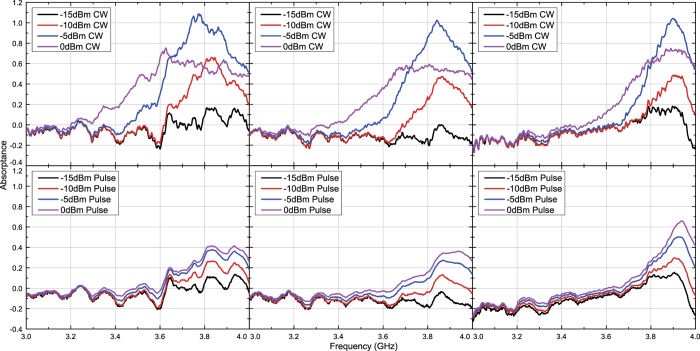
Measured absorptance of inductor-based metasurface for TE polarized waves. The incident angle was set to 20 (left), 40 (centre) and 60 degrees (right). The top and bottom panels respectively represent the absorptances for CWs and short pulses (50 ns long). See Supplementary Information for the difference between CW and pulse absorptances.

**Figure 13:**
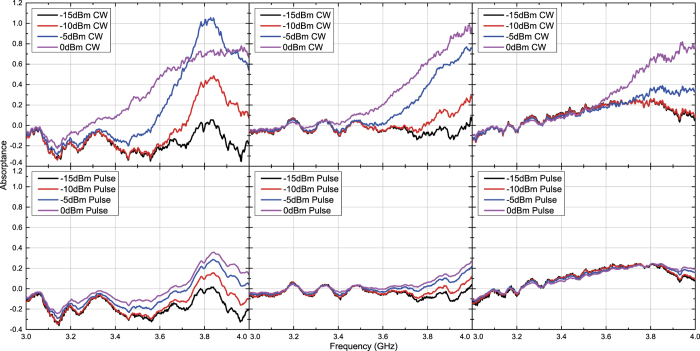
Measured absorptance of inductor-based metasurface for TM polarized waves. The incident angle was set to 20 (left), 40 (centre) and 60 degrees (right). The top and bottom panels respectively represent the absorptances for CWs and short pulses (50 ns long). See Supplementary Information for the difference between CW and pulse absorptances.

**Figure 14:**
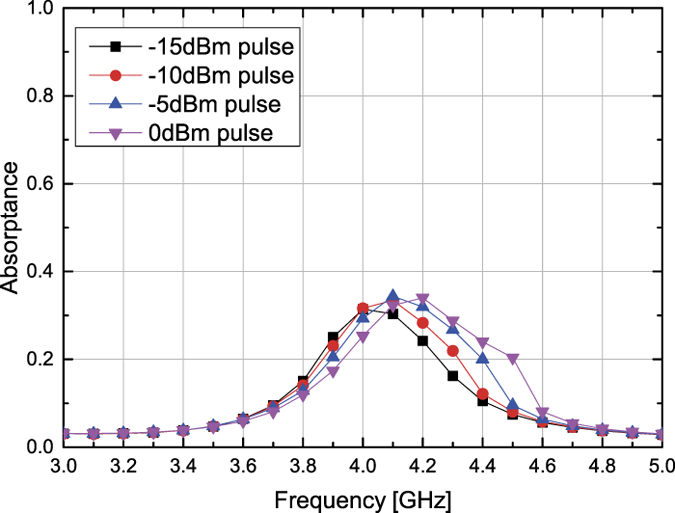
Simulated absorptance of inductor-based metasurface for TM polarized waves with a larger inductance. Compared to Fig. 11, the inductance (and time constant) was increased by a factor of ten (i.e. to 1 mH).

Basically, the same angle dependence is obtained for inductor-based metasurfaces if the structures are properly designed including their time constants, which play a very important role in determining the response of waveform selectivity. For example, in Figs 10, 11, 12, 13 the inductance and resistance values were respectively set to 100 *μ*H and 5.5 Ω (the resonant frequency of the inductor package was 10 MHz). Under this circumstance the inductor-based metasurfaces more strongly absorbed CWs than short pulses but with reduced difference compared to that of the capacitor-based metasurfaces demonstrated in Figs 3, 4, 8 and 9 (e.g. compare the bottom left panel of Fig. 11 to the top left panel of Fig. 4). This is because the time constant used was not large enough so that the contrast between them was weakened. In Fig. 14 the same structure was simulated with a TM pulsed wave at 20 degrees but with the inductance increased by a factor of ten so that the absorptance was decreased from approximately 0.55 to 0.35.

## Conclusion

We have demonstrated how two types of waveform-selective metasurfaces, specifically, capacitor-based metasurfaces and inductor-based metasurfaces vary their absorbing performances with various incident angles. Capacitor-based metasurfaces were both numerically and experimentally shown to absorb short pulses more effectively than CWs at the same frequency, while inductor-based metasurfaces more absorbed CWs than short pulses. These waveform selectivities were reduced for a large incident angle, which was improved by properly designing the metasurfaces including materials properties, time constants and input power. These results are expected to be useful for applying waveform-selective metasurfaces for a wide range of conventional electromagnetic devices and applications, which gives us an additional degree of freedom to control their functionalities (e.g. waveform-selective wireless communications^26^).

## Additional Information

**How to cite this article**: Wakatsuchi, H. *et al*. Responses of Waveform-Selective Absorbing Metasurfaces to Oblique Waves at the Same Frequency. *Sci. Rep.* **6**, 31371; doi: 10.1038/srep31371 (2016).

## Supplementary Material

Supplementary Information

## Figures and Tables

**Figure 1:**
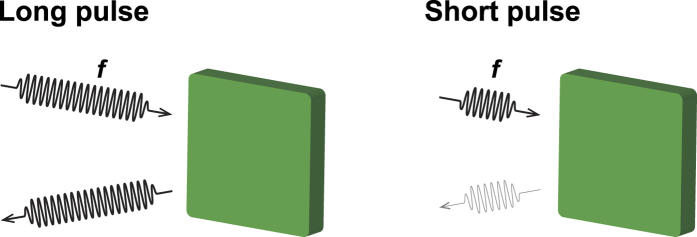
Waveform selectivity. Waveform-selective metasurfaces enable us to distinguish different waves even at the same frequency in response to their waveforms or pulse widths.

**Figure 2:**
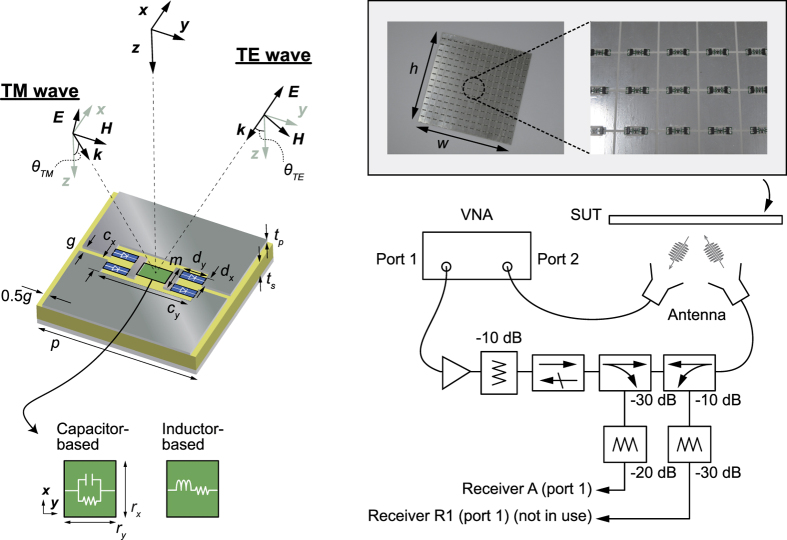
Model, sample and method. (Left) A periodic unit of a metasurface was simulated with periodic boundaries that were applied for *x*- and *y*-axis directions to effectively model an infinite array of the periodic structure. Each dimension was given from *c*_*x*_ = 1.7, *c*_*y*_ = 7.6, *d*_*x*_ = 0.5, *d*_*y*_ = 1.3, *g* = 1.0, *l* = 2.4, *p* = 18.0, *r*_*x*_ = 2.0, *r*_*y*_ = 1.0, *t*_*p*_ = 0.017 and *t*_*s*_ = 1.524 (all in mm). The capacitor-based metasurface had a capacitor (1 nF) in parallel with a resistor (10 kΩ), while the inductor-based metasurface had an inductor (100 *μ*H) in series with a resistor (5.5 Ω). (Right) The measurement was performed using a measurement setup including a VNA that enabled us to control the pulse width of the incoming wave. The dimensions of samples were all *h* = *w* = 270 mm (corresponding to 15 periodic units). Antennas were located at 400 mm away from the samples.
