# Supplementary material for: Responses of Waveform-Selective Absorbing Metasurfaces to Oblique Waves at the Same Frequency
Source: Sci Rep. 2016 Aug 12;6:31371. doi: 10.1038/srep31371 (PMC4981871; doi:10.1038/srep31371)
Supplement: Supplementary Information [file srep31371-s1.pdf]

Supplementary Information

Responses of Waveform-Selective Absorbing Metasurfaces to Oblique Waves at the Same Frequency

Hiroki Wakatsuchi, Fei Gao, Satoshi Yagitani and Daniel F. Sievenpiper

- **Difference between pulse absorptance and CW absorptance**

Difference between pulse absorptance and CW absorptance

Figs. 3, 4 and 8 to 13 showed absorptances of capacitor- and inductor-based metasurfaces. The differences between CWs and pulses (50 ns) are plotted in Figs. 15 to 18.

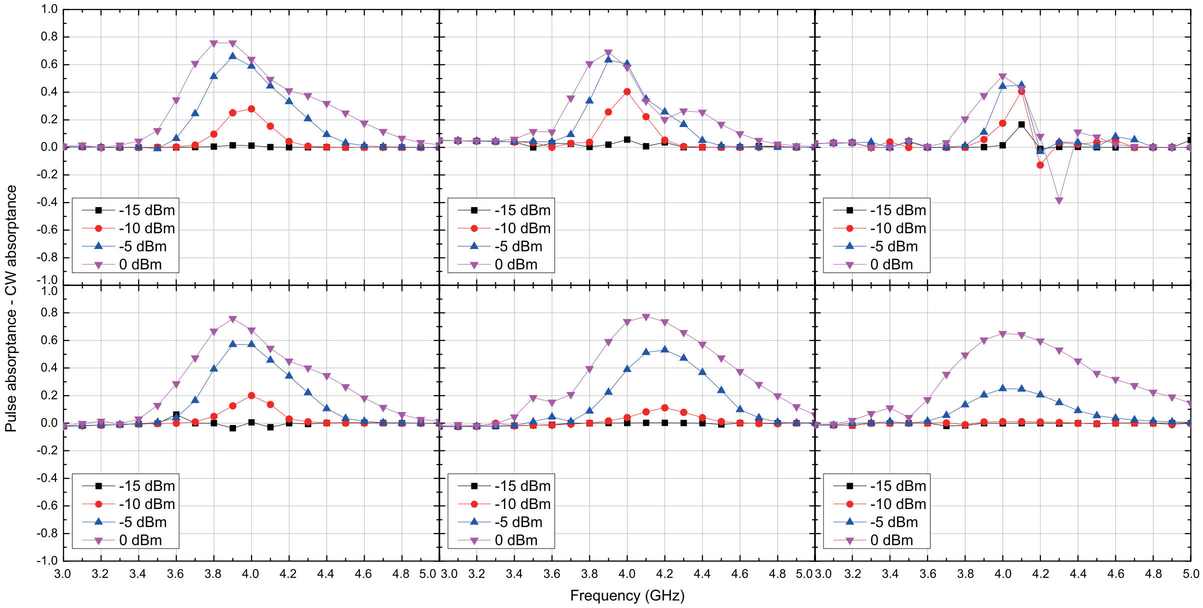

Figure 15. Differences in simulated absorptance of capacitor-based metasurface. The incident angle was set to 20 (left), 40 (centre) and 60 degrees (right). The top and bottom panels respectively represent the differences in TE and TM waves.

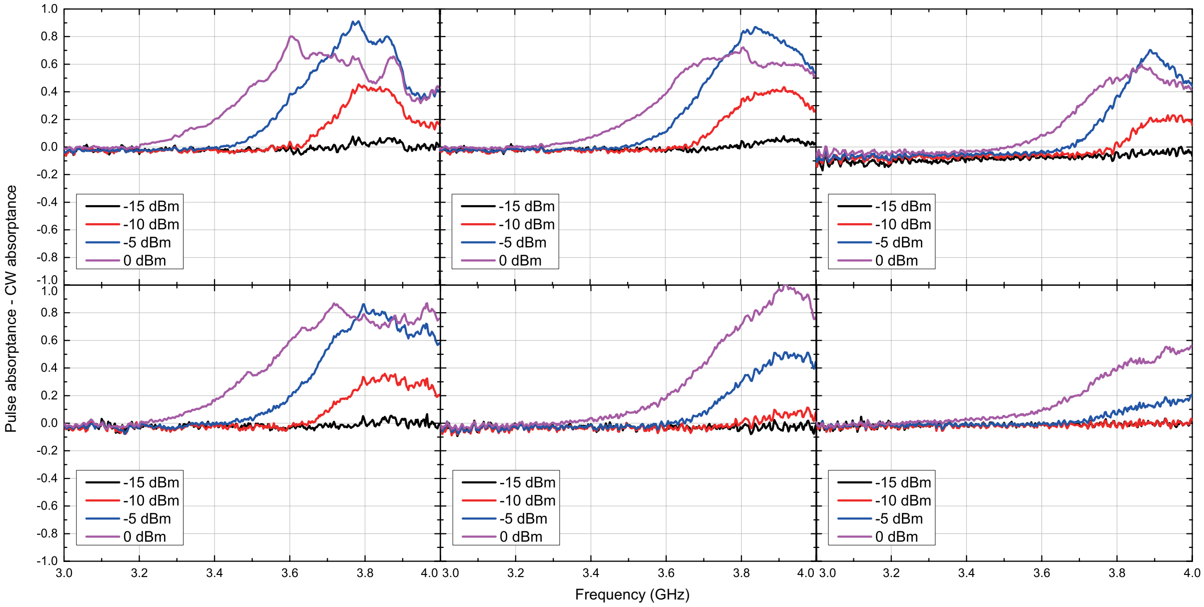

Figure 16. Differences in measured absorptance of capacitor-based metasurface. The incident angle was set to 20 (left), 40 (centre) and 60 degrees (right). The top and bottom panels respectively represent the differences in TE and TM waves.

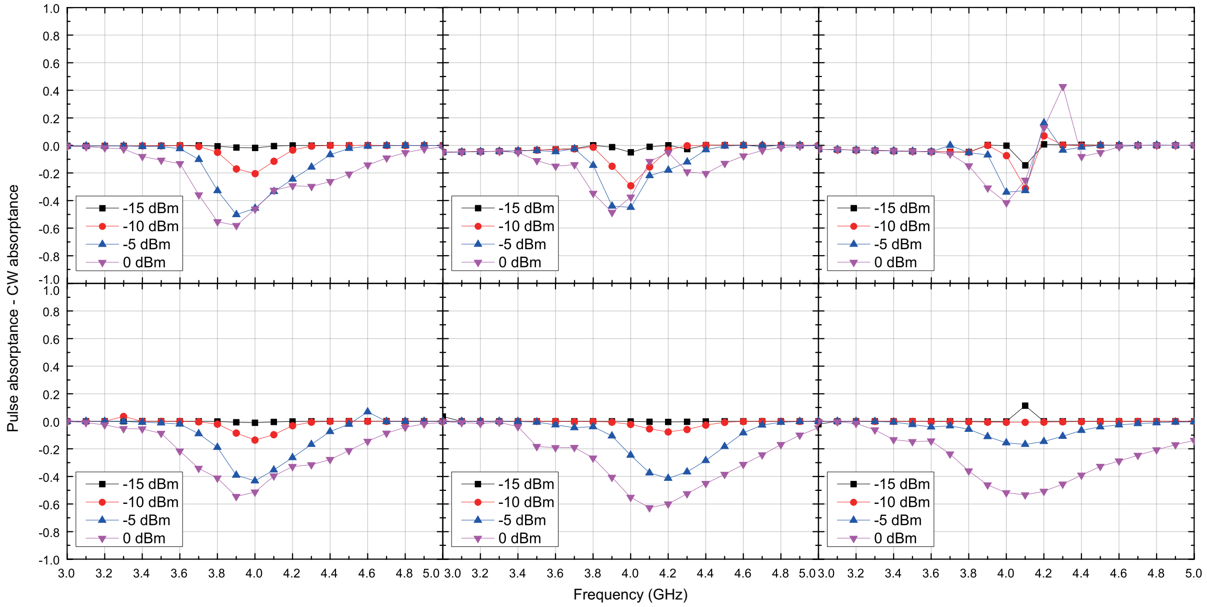

Figure 17. Differences in simulated absorptance of inductor-based metasurface. The incident angle was set to 20 (left), 40 (centre) and 60 degrees (right). The top and bottom panels respectively represent the differences in TE and TM waves.

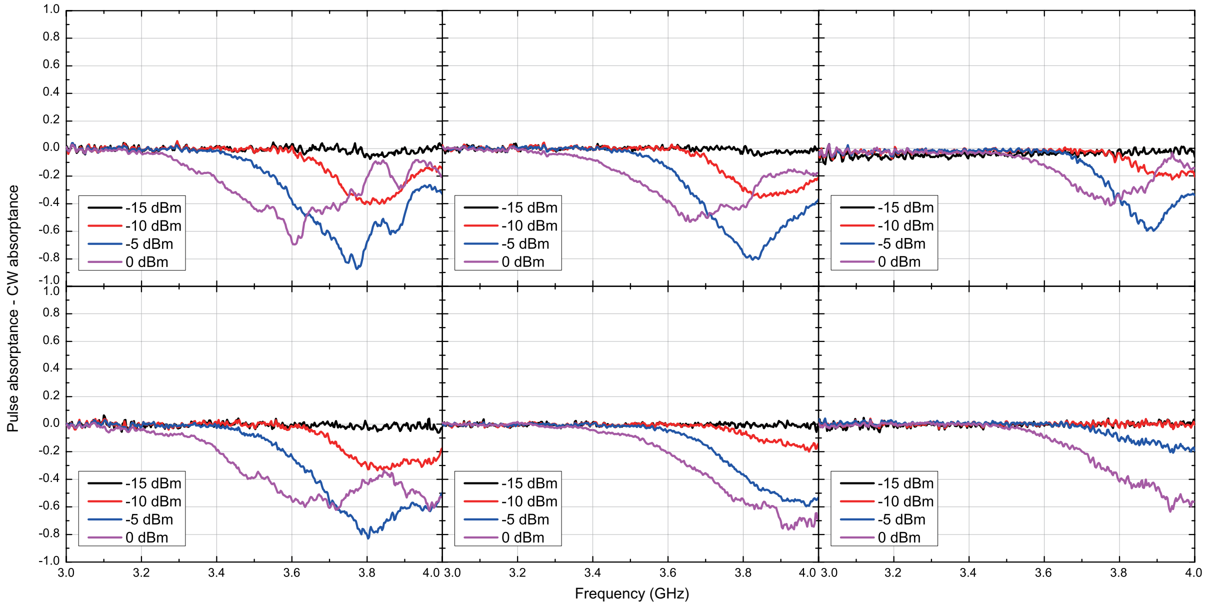

Figure 18. Differences in measured absorptance of inductor-based metasurface. The incident angle was set to 20 (left), 40 (centre) and 60 degrees (right). The top and bottom panels respectively represent the differences in TE and TM waves.
